# Development and application of a nomogram model for the prediction of carbapenem-resistant *Klebsiella pneumoniae* infection in neuro-ICU patients

**DOI:** 10.1128/spectrum.03096-23

**Published:** 2023-12-07

**Authors:** Guangyu Lu, Jingyue Zhang, Tian Shi, Yuting Liu, Xianru Gao, Qingping Zeng, Jiali Ding, Juan Chen, Kai Yang, Qiang Ma, Xiaoguang Liu, Chuanli Ren, Hailong Yu, Yuping Li

**Affiliations:** 1 School of Public Health, Medical College of Yangzhou University, Yangzhou University, Yangzhou, China; 2 School of Nursing, Medical College of Yangzhou University, Yangzhou University, Yangzhou, China; 3 Neuro-Intensive Care Unit, Department of Neurosurgery, Clinical Medical College, Yangzhou University, Yangzhou, China; 4 College of Information Engineering, Yangzhou University, Yangzhou, China; 5 Department of Laboratory Medicine, Clinical College of Yangzhou University, Yangzhou, China; 6 Department of Neurology, Northern Jiangsu People’s Hospital, Yangzhou, China; 7 Department of Neuro-Intensive Care Unit, Clinical Medical College of Yangzhou University, Yangzhou, China; Quest Diagnostics Nichols Institute, Chantilly, Virginia, USA

**Keywords:** nosocomial infection, carbapenem-resistant *Klebsiella pneumoniae* infection, prediction model, nomogram, neuro-ICU patients

## Abstract

This study aimed to develop and validate a simple-to-use nomogram model to assess the risk of infection caused by carbapenem-resistant *Klebsiella pneumoniae* (CRKP) in neurocritically ill patients. The clinical data of 544 patients with CRKP infection admitted to a neurointensive care unit (neuro-ICU) were retrospectively analyzed. The demographic data, laboratory test results, and clinical characteristics of patients in the neuro-ICU were collected. Subsequently, multivariate regression analysis was used to construct a nomogram to predict the risk of CRKP infection in these patients. The calibration ability, clinical effectiveness, and discriminative ability of the nomogram were evaluated. The incidence of CRKP infection was estimated to be 6.43%, and a majority of bacterial isolates causing the infection were found in sputum (74.3%). Multivariate regression analysis showed that the number of antibiotics of ≥2 [odds ratio (OR): 9.08, 95% confidence interval (CI): 2.78–29.71], undergoing surgery (OR: 3.84, 95% CI: 1.09–13.54), and long neuro-ICU stay (OR: 1.08, 95% CI: 1.01–1.14) were associated with CRKP infection in neurocritically ill patients. The nomogram model demonstrated good calibration and discrimination in both the training and validation sets, with area under the curve values of 0.860 and 0.907, respectively. This study developed and validated a nomogram that combines three easily accessed variables during clinical practice to predict the risk of nosocomial CRKP infection in neuro-ICU patients. The tool demonstrated a good predictive performance and discrimination, which might serve as a useful tool to facilitate early detection and reduction of the CRKP infection risk in neurocritically ill patients.

**IMPORTANCE:**

Patients in neuro-ICU are at a high risk of developing nosocomial CRKP infection owing to complex conditions, critical illness, and frequent invasive procedures. However, studies focused on constructing prediction models for assessing the risk of CRKP infection in neurocritically ill patients are lacking at present. Therefore, this study aims to establish a simple-to-use nomogram for predicting the risk of CRKP infection in patients admitted to the neuro-ICU. Three easily accessed variables were included in the model, including the number of antibiotics used, surgery, and the length of neuro-ICU stay. This nomogram might serve as a useful tool to facilitate early detection and reduction of the CRKP infection risk of neurocritically ill patients.

## INTRODUCTION

*Klebsiella pneumoniae* is a drug-resistant bacterial pathogen that is responsible for causing nosocomial infections (1). The excessive use of antibiotics in recent years has resulted in a remarkable rise in the incidence of carbapenem-resistant *Klebsiella pneumoniae* (CRKP) globally (2 – 5). CRKP has been identified as a critical priority pathogen that poses a significant threat to human health (5). According to recent studies, Greece has the highest detection rate of CRKP at 54.9%, followed by Eastern Europe at 22.5%, Argentina at 14%, and the Philippines at 11.0% (6). The China Antimicrobial Resistance Surveillance System reported an increasing annual detection rate of CRKP from 6.4% in 2014 to 11.3% in 2021 (7). The resistance rates of *K. pneumoniae* to imipenem and meropenem have significantly increased over the years. In 2005, the resistance rates were 3.0% and 2.9%, respectively, whereas in 2022, they have risen to 26.0% and 27.5% (7). Several studies have reported that the mortality rate of patients infected with CRKP is approximately 40%–50% (8, 9). Patients with CRKP infection, especially those with bloodstream infection, have a mortality rate of up to 70% (2, 9). Therefore, the identification and prevention of CRKP infection at an early stage are significant challenges for healthcare providers and international infection control programs (10).

CRKP is a major concern for critically ill patients due to its high morbidity and mortality rates (11). Surveillance data have shown that the prevalence of CRKP among patients admitted to the intensive care unit (ICU) ranges from 20.8% to 48.1%, which is notably higher than that observed in other clinical departments (11, 12). Additionally, it is important to note that the mortality rates of patients infected with CRKP in the ICU are significantly higher compared to those in non-ICU settings (13). Numerous studies have investigated the risk factors associated with CRKP infection in patients admitted to the ICU, resulting in the development of various risk prediction models (14 – 17). However, there is growing recognition that the critically ill patient is a highly heterogeneous population and that using the one-size-fits-all approach in the ICU may lead to inconsistent results (18, 19). Therefore, establishing a single risk prediction model for all critically ill patients may largely limit the application of the model in specific groups of critically ill patients, such as neurocritically ill patients.

The neurointensive care unit (neuro-ICU) is a specialized and personalized intensive care unit that provides multidisciplinary clinical care to patients who are critically ill due to neurological and neurosurgical issues (20). Patients in the neuro-ICU are at a high risk of developing nosocomial CRKP infection owing to their complex and critical conditions and frequent invasive procedures (21). Moreover, researchers have demonstrated higher mortality among CRKP-infected neuro-ICU patients than among CRKP-infected ICU patients (22, 23). However, limited research has been conducted on risk factors and prediction models for CRKP infection in neuro-ICU patients. Very few studies have investigated their expectations and difficulties in implementing such predictions in their clinical practice. Therefore, this study aimed to develop and validate a nomogram model to predict the risk of CRKP infection in neurocritically ill patients and to provide information regarding the expectations and challenges associated with the use of this model in clinical settings.

## MATERIALS AND METHODS

### Study population

This retrospective cohort study was conducted from January to December 2019 at a tertiary hospital in China. The subjects were patients admitted to the neuro-ICU, and the study included only the first positive culture of CRKP from each patient as a sample. Patients aged ≥18 years were included. The exclusion criteria were as follows: (i) stay in the neuro-ICU for <24 hours, (ii) detection of CRKP during the first 48 hours in the neuro-ICU or 48 hours after leaving the neuro-ICU, (iii) colonization by CRKP without any clinical symptoms, (iv) incomplete clinical data, and (v) repeat check-in to neuro-ICU.

### Diagnosis of CRKP infection

The *K. pneumoniae* strain, initially isolated from a clinical specimen, was used to define the index culture. Subsequently, the nosocomial infection was confirmed based on clinical signs and symptoms, imaging reports, and easily obtained clinical indicators, which helped to distinguish the infection from colonization (24). Acquired nosocomial infection was defined as an infectious disease acquired after 48 hours of hospitalization (25). The CRKP strains were resistant to at least one class of carbapenem antibiotic (imipenem, meropenem, or ertapenem) (26).

### Antimicrobial susceptibility testing

Matrix-assisted laser desorption/ionization time-of-flight mass spectrometry (bioMerieux, France) was used for the identification of bacteria. The VITEK-2 Compact (bioMérieux) automatic microbial identification instrument and its accompanying XN04 and N335 cards were used for the determination of drug sensitivity MIC values. *Escherichia coli* ATCC25922 and *Pseudomonas aeruginosa* ATCC27853 were used as benchmark strains for testing drug susceptibility. To avoid double counting, only the first isolate according to the ID number was recorded for each patient.

### Data collection

The electronic medical records of patients were extracted from the hospital health information system, and the results of antimicrobial susceptibility testing were obtained from a hospital affiliated microbiology laboratory center. The study collected data on various aspects of the patients including their general information such as age, sex, comorbidities, and the length of their stay in neuro-ICU. Information on admission was also recorded along with laboratory test results such as the levels of albumin, serum creatinine, and total protein within 48 hours of admission. In addition, the study also documented whether the patient had undergone surgery and the specific number of antibiotics that were administered. See Table S1 for more details regarding variable definition.

### Statistical analysis

Categorical variables were presented as counts and percentages (%), while continuous variables were presented as either mean ± standard deviation or median and interquartile range. The independent samples *t*-test was used to compare two groups of parametric values, and the Mann-Whitney *U* test was used to compare two groups of non-parametric values. Categorical variables were compared using the chi-square test. Following the univariate analysis, we conducted multivariate logistic regression analysis to determine the odds ratios (ORs) and 95% confidence intervals (CIs) of independent variables. Variables for the multivariate model were selected based on their physiological relevance and statistical significance in the univariate analysis. A threshold *P* value of 0.25 was used in the selection process. All statistical tests were two-tailed, and a significance level of 0.05 was used for the multivariate analysis. The statistical tests were conducted using SPSS Statistics software (version 26, USA).

A predictive nomogram was constructed through multivariate analysis in the training set. Multivariate analysis was performed to evaluate the predictive models for three indicators: discriminative power, calibration power, and clinical effectiveness. Receiver operating characteristic (ROC) curves were plotted, and the area under the curve (AUC) values were estimated to evaluate the discriminative power of the nomogram. Calibration curves were plotted to evaluate the predictive accuracy of the nomogram. To assess the clinical effectiveness, we employed decision curve analysis and clinical impact curve analysis. In both the development and validation stages, statistical tests were conducted using R statistical software (version 4.2.2).

### Logic behind the nomogram

The nomogram integrates multiple predictive variables based on multivariate logistic regression analysis and uses line segments with scale to represent the relationship between variables in the prediction model (27). The length of the line segment corresponding to each variable in the nomogram is converted from the regression coefficient (28). The length of the line segment reflects the contribution of the variable to the outcome, and the scale on the line segment represents the value range of the variable (28). The values of patients are placed on each variable axis, and a line is drawn upward to determine the score of each variable corresponding to the first row of “points.” The total score is obtained by adding the single scores corresponding to the “total points” row, and a line is drawn down the probability axis to determine the risk of CRKP infection.

## RESULTS

### Development and validation of the prediction tool for CRKP infection in neuro-ICU

**Fig 1:**
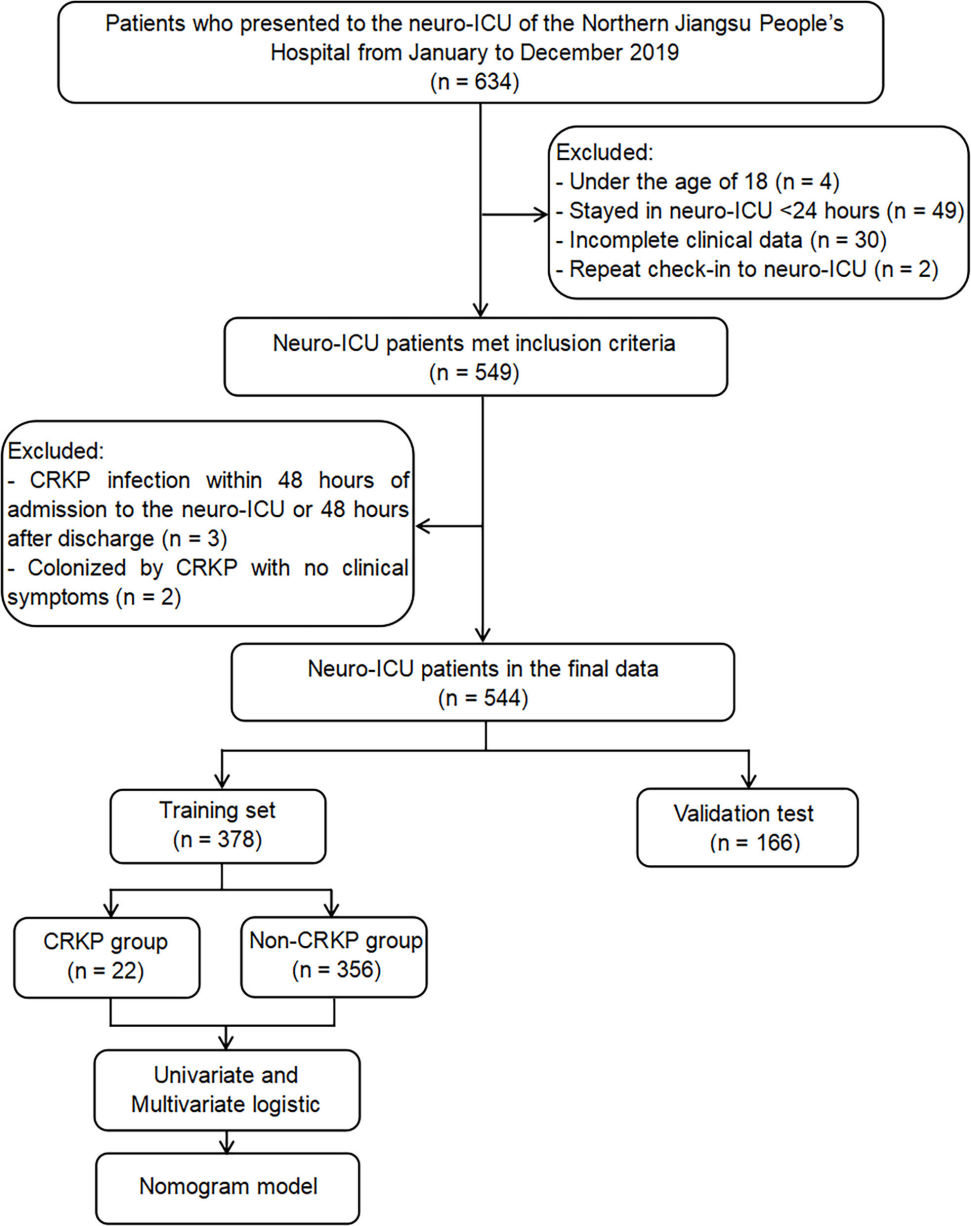
Study flow diagram.

A total of 634 patients were admitted to the neuro-ICU between January and December 2019. Of these 634 patients, 49 patients stayed in the neuro-ICU for <24 hours; 30 patients had missing data; 4 patients were below 18 years of age; 3 patients were found to have an infection within 48 hours of admission to the neuro-ICU or 48 hours after discharge; 2 patients with CRKP underwent colonization; and 2 patients were readmitted. The study included 544 patients who met the inclusion criteria (Fig. 1). Table 1 presents a concise overview of the primary demographic and clinical features of the participants.

**TABLE 1:** Demographic and clinical characteristics of the neuro-ICU patients^*a*^

Variable	Total	Training set	Validation set
	(*n* = 544)	(*n* = 378)	(*n* = 166)
Infection rate, *n* (%)	35 (6.4)	22 (5.8)	16 (9.6)
Clinical parameters			
Age (year), mean (SD)	60.3 (13.1)	60.6 (13.2)	59.5 (12.8)
Male sex, *n* (%)	331 (60.8)	229 (60.6)	102 (61.4)
History of anticoagulants, *n* (%)	25 (4.6)	16 (4.2)	9 (5.4)
Length of stay in neuro-ICU (days), median (IQR)	5.0 (2.0–10.0)	4.0 (2.0–10.0)	5.0 (2.0–10.0)
Diabetes, yes (%)	78 (14.3)	52 (13.8)	26 (15.7)
Hypertension, yes (%)	257 (47.2)	174 (46)	83 (50)
Heart disease, yes (%)	27 (5)	17 (4.5)	10 (6)
Cerebral infarction, yes (%)	50 (9.2)	41 (10.8)	9 (5.4)
Chronic renal insufficiency, yes (%)	11 (2)	10 (2.6)	1 (0.6)
Admission season, *n* (%)			
Spring	127 (23.3)	90 (23.8)	37 (22.3)
Summer	141 (25.9)	95 (25.1)	46 (27.7)
Autumn	169 (31.1)	114 (38.1)	55 (33.1)
Winter	107 (19.7)	79 (20.9)	28 (16.9)
Admission diagnosis, *n* (%)			
Cerebral hemorrhage	268 (49.3)	178 (47.1)	90 (54.2)
Brain injury	144 (26.5)	110 (29.1)	40 (24.1)
Aneurysm	88 (16.2)	59 (15.6)	29 (17.5)
Epilepsy	31 (5.7)	21 (5.6)	10 (6.0)
Brain tumor	13 (2.4)	10 (2.6)	3 (1.8)
Laboratory parameters			
INR, median (IQR)	1.05 (1–1.13)	1.05 (1.00–1.12)	1.05 (1.00–1.13)
BMI (kg/m^2^)	23.9 (3.8)	23.9 (3.9)	24.1 (3.5)
Albumin (g/L)	40.9 (6.8)	40.5 (7.0)	41.7 (6.4)
Total protein, (g/L), median (IQR)	70.10 (62.10–76.28)	69.30 (60.60–75.60)	71.40 (64.90–77.30)
RBC (10^12^/L）	4.1 (0.8)	4.1 (0.9)	4.3 (0.7)
WBC (10^9^/L), median (IQR)	12.17 (9.20–16.48)	12.10 (9.18–16.53)	12.36 (9.25–16.24)
Platelets (10^9^/L), median (IQR)	167.0 (119.0–218.0)	164.0 (119.8–211.3)	174.0 (118.0–224.0)
Hemoglobin (g/L)	125.3 (29.0)	123.8 (30.7)	129 (24.5)
Serum creatinine (μmol/L), median (IQR)	65.00 (54.00–82.98)	69.95 (55.65–83.00)	63.80 (52.45–81.00)
Procalcitonin (ng/mL), median (IQR)	0.29 (0.10–1.08)	0.30 (0.10–1.19)	0.28 (1.00–0.88)
ALT (U/L), median (IQR)	26.5 (17.0–36.8)	26.0 (18.0–36.0)	27.5 (17.0–38.0)
AST (U/L), median (IQR)	32.5 (25.0–46.8)	33.0 (24.0–47.0)	31.5 (25.0–42.0)
Uric acid (μmol/L)	294.4 (109.7)	294.5 (112.5)	294.2 (103.4)
Urea (mmol/L), median (IQR)	5.4 (4.24–6.59)	5.22 (4.22–6.43)	5.69 (4.39–6.91)
LAC (mmol/L)	3.2 (2.9)	3.2 (3.2)	3.1 (1.9)
Glucose (mmol/L), median (IQR)	8.6 (6.72–11.22)	8.6 (6.59–11.10)	8.90 (6.89–11.40)
Systolic pressure (mmHg)	153.8 (37.9)	152.4 (37.4)	157 (39.0)
Treatment			
Surgery, yes (%)	320 (58.8)	225 (59.5)	95 (57.2)
Number of antibiotics, ≥2 (%)	93 (17.1)	62 (16.4)	31 (18.7)

^*a*^ The quantitative data are distributed normally, expressed by means ± standard deviations; the quantitative data are skewed and expressed as medians (25th–75th percentile). Qualitative data are expressed in *n* (%). Admission season: spring (1 March–31 May), summer (1 June–31 August), autumn (1 September–30 November), and winter (1 December–28 February). ALT, alanine transaminase; AST, aspartate transaminase; BMI, body mass index; INR, international normalized ratio; IQR, interquartile range; LAC, lactic acid; RBC, red blood cell; SD, standard deviation; WBC, white blood cell.

### Incidence of CRKP infection

The overall incidence of CRKP infection was 6.43% (35 of 544), with an incidence of 5.82% (22 of 378) in the training set and 7.83% (13 of 166) in the validation set. Among the CRKP strains, 26 (74.3%) were isolated from sputum samples and 9 (25.7%) from urine samples.

### Results of univariate and multivariate analyses

In the univariate analysis, admission season (*P* = 0.234), LAC levels (*P* = 0.233), urea levels (*P* = 0.198), sex (*P* = 0.108), platelets levels (*P* = 0.07), surgery (*P* = 0.037), serum creatinine levels (*P* = 0.13), length of stay in neuro-ICU (*P* < 0.001), and number of antibiotics (*P* < 0.001) were significantly associated with CRKP infection in the neuro-ICU. Multicollinearity test based on the variance inflation factor (VIF) showed no evidence of collinearity among the explanatory variables. The multivariable analysis results revealed that a longer stay in the neuro-ICU (OR: 1.08, 95% CI: 1.01–1.14, *P* = 0.017), number of antibiotics of ≥2 (OR: 9.08, 95% CI: 2.78–29.71, *P* < 0.001), and surgery (OR: 3.84, 95% CI: 1.09–13.54, *P* = 0.036) were independent risk factors for contracting CRKP infection (Table 2).

**TABLE 2:** Multivariate analysis of risk factors for CRKP neuro-ICU inpatients^*a*^

Variable	*β*	SE	Wald	OR (95% Cl)	*P* value
Number of antibiotics ≥2	2.206	0.605	13.315	9.082 (2.777–29.707)	<0.001
Length of stay in neuro-ICU	0.072	0.03	5.706	1.075 (1.013–1.140)	0.017
Surgery	1.346	0.643	4.381	3.840 (1.089–13.538)	0.036

^*a*^ *β* is the regression coefficient. *P* was set at <0.05 for the multivariable results. Variables were excluded based on univariate analysis (*P* < 0.25): age (*P* = 0.971), diabetes (*P* = 0.537), hypertension (*P* = 0.620), cerebral infarction (*P* = 0.785), heart disease (*P* = 0.991), admission diagnosis (*P* = 0.801), chronic renal insufficiency (*P* = 0.573), systolic pressure (*P* = 0.317), history of anticoagulants (*P* = 0.940), albumin (*P* = 0.444), BMI (*P* = 0.298), total protein (*P* = 0.667), RBC (*P* = 0.579), WBC (*P* = 0.869), hemoglobin (*P* = 0.376), procalcitonin (*P* = 0.839), ALT (*P* = 0.904), AST (*P* = 0.772), uric acid (*P* = 0.857), glucose (*P* = 0.867), and INR (*P* = 0.883). Variables were excluded in the final multivariable analysis (*P* < 0.05): gender (*P* = 0.203), serum creatinine (*P* = 0.210), platelets (*P* = 0.316), LAC levels (*P* = 0.161), urea levels (*P* = 0.418), and admission season (*P* = 0.394). CI, confidence interval; OR, odds ratio; SE, standard error.

### Development of the nomogram

**Fig 2:**
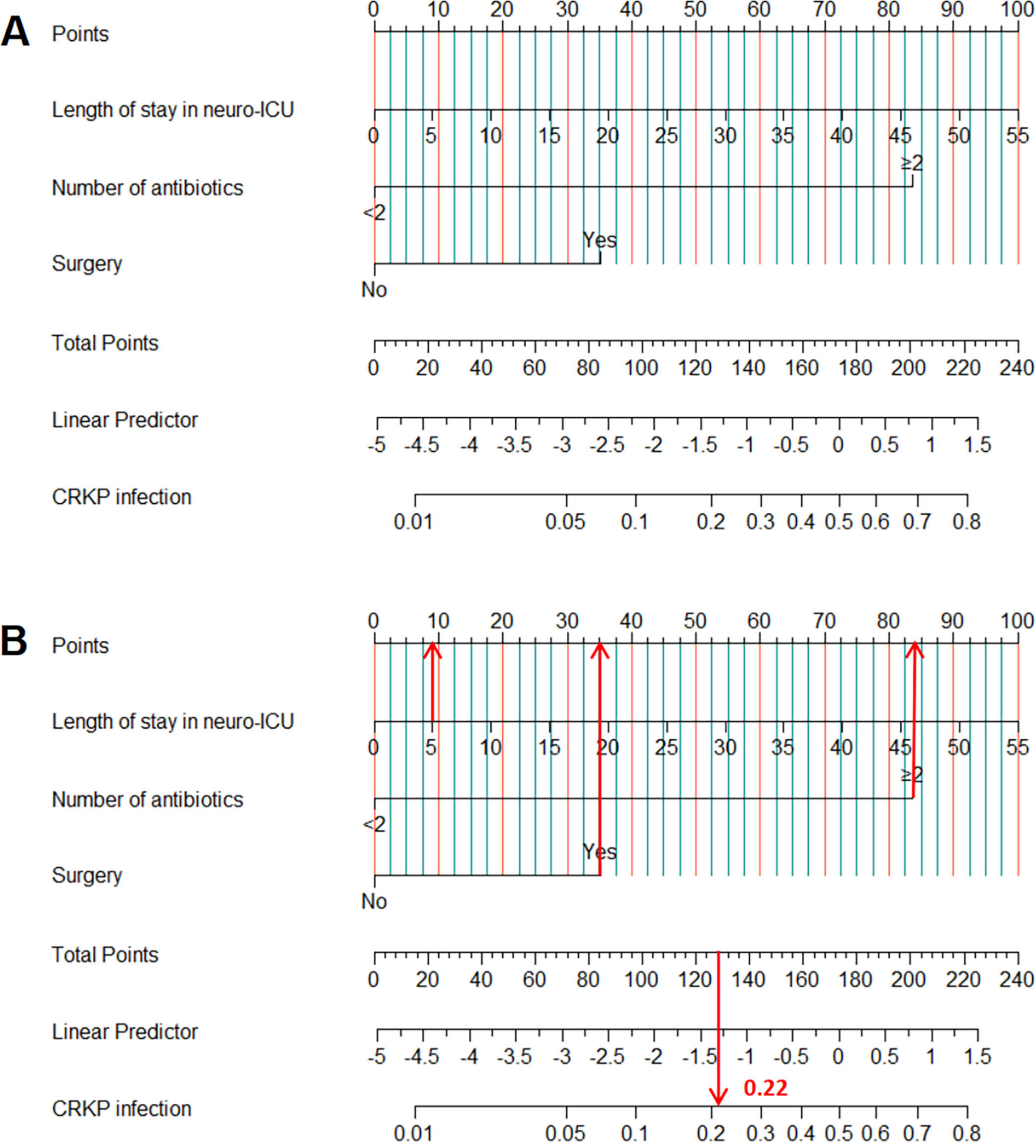
(A) Nomogram to predict the risk of CRKP infection in neuro-ICU patients (B) Example of an application of the nomogram to predict the occurrence of CRKP infection in neuro-ICU patients.

In this study, the nomogram model was developed to predict CRKP infection in neuro-ICU patients based on three variables: length of stay in neuro-ICU, number of antibiotics (≥2 or <2), and surgery (yes, no). The sex (*P* = 0.203), serum creatinine levels (*P* = 0.210), admission season (*P* = 0.394), platelets levels (*P* = 0.316), urea levels (*P* = 0.418), and LAC levels (*P* = 0.161) were excluded from the model. The relationship between total score and risk of CRKP infection is illustrated in Fig. 2A. Additionally, Fig. 2B demonstrates how the nomogram can be used to predict the risk of CRKP infection in patients admitted to the neuro-ICU .

### Validation of the nomogram

**Fig 3:**
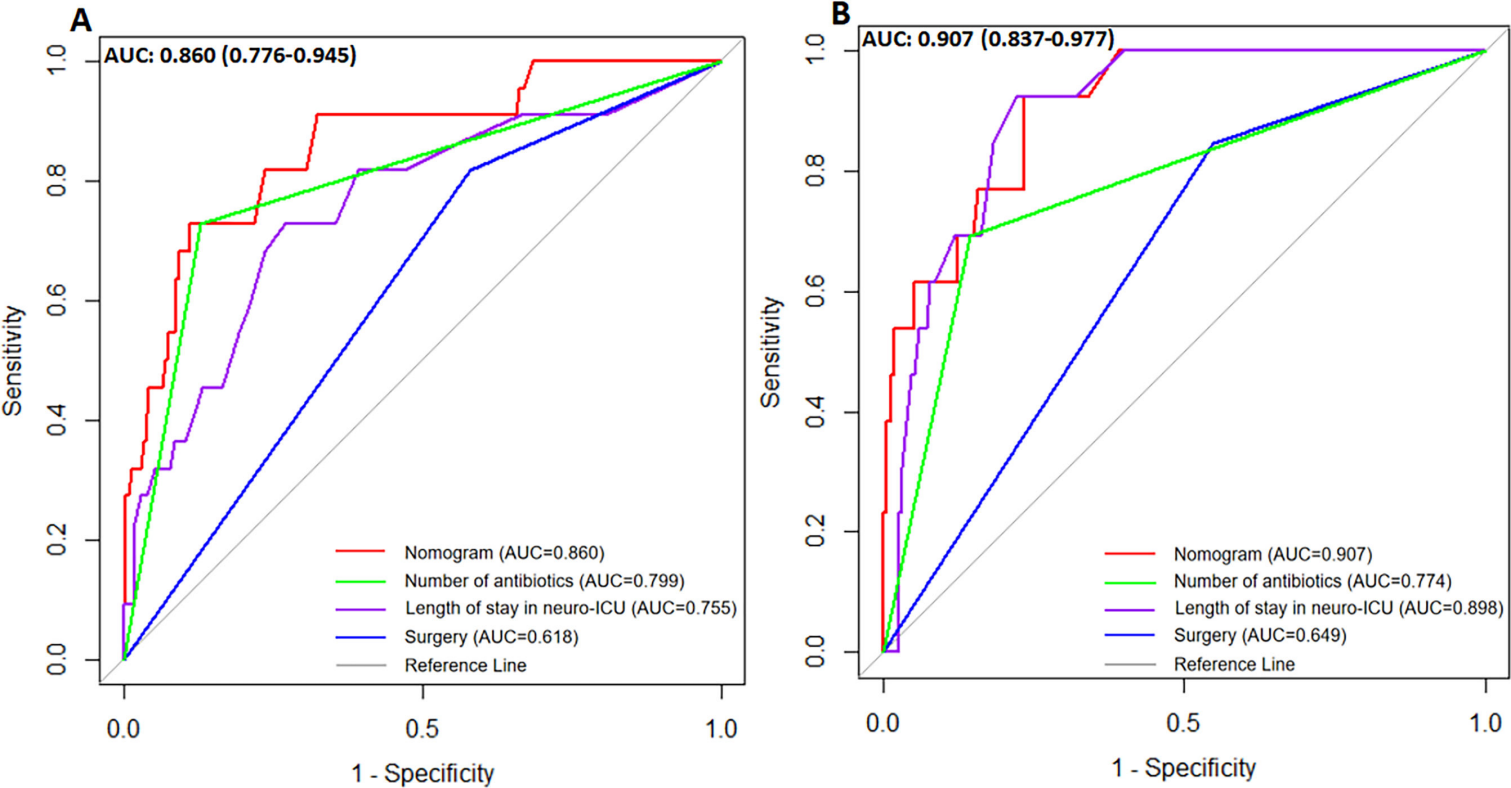
(A) The ROC curves of the nomogram model in the training set. (B) The ROC curves of the nomogram model in the validation set.

**Fig 4:**
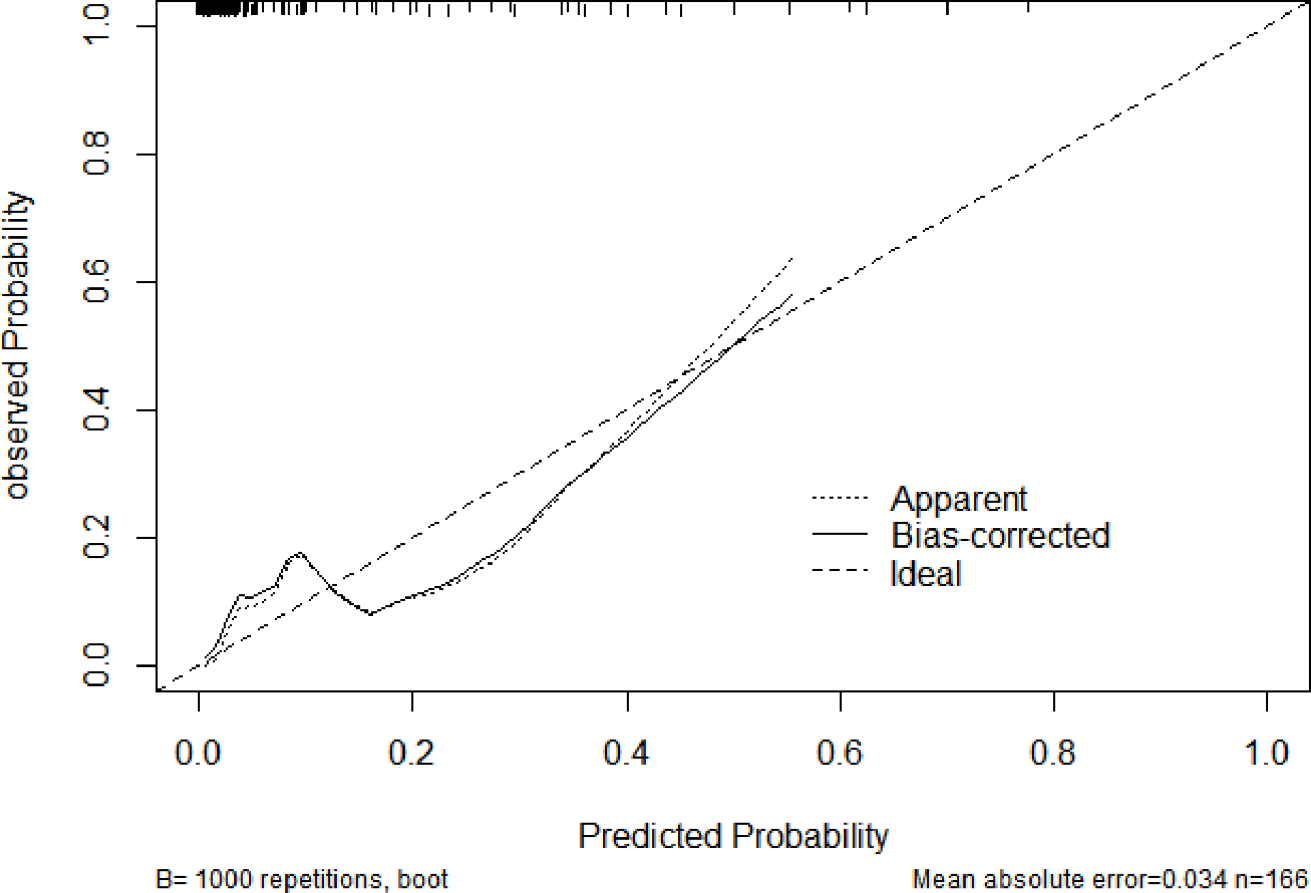
The calibration curve of the nomogram model in the validation set.

The nomogram model exhibited a good discriminative ability, as evidenced by its AUC of 0.860 in the training set and 0.907 in the validation set. The ROC curves for both sets indicated that the predictive accuracy of the nomogram was superior to that of individual risk factors (Fig. 3). In addition to validation, we also recorded the calibration curves of the nomogram in the validation set and observed a mean absolute error of 0.034. This indicates that there was good predictive agreement and calibration between predictions and actual probability for the nomogram, as shown in Fig. 4.

### Clinical applicability of the nomogram

**Fig 5:**
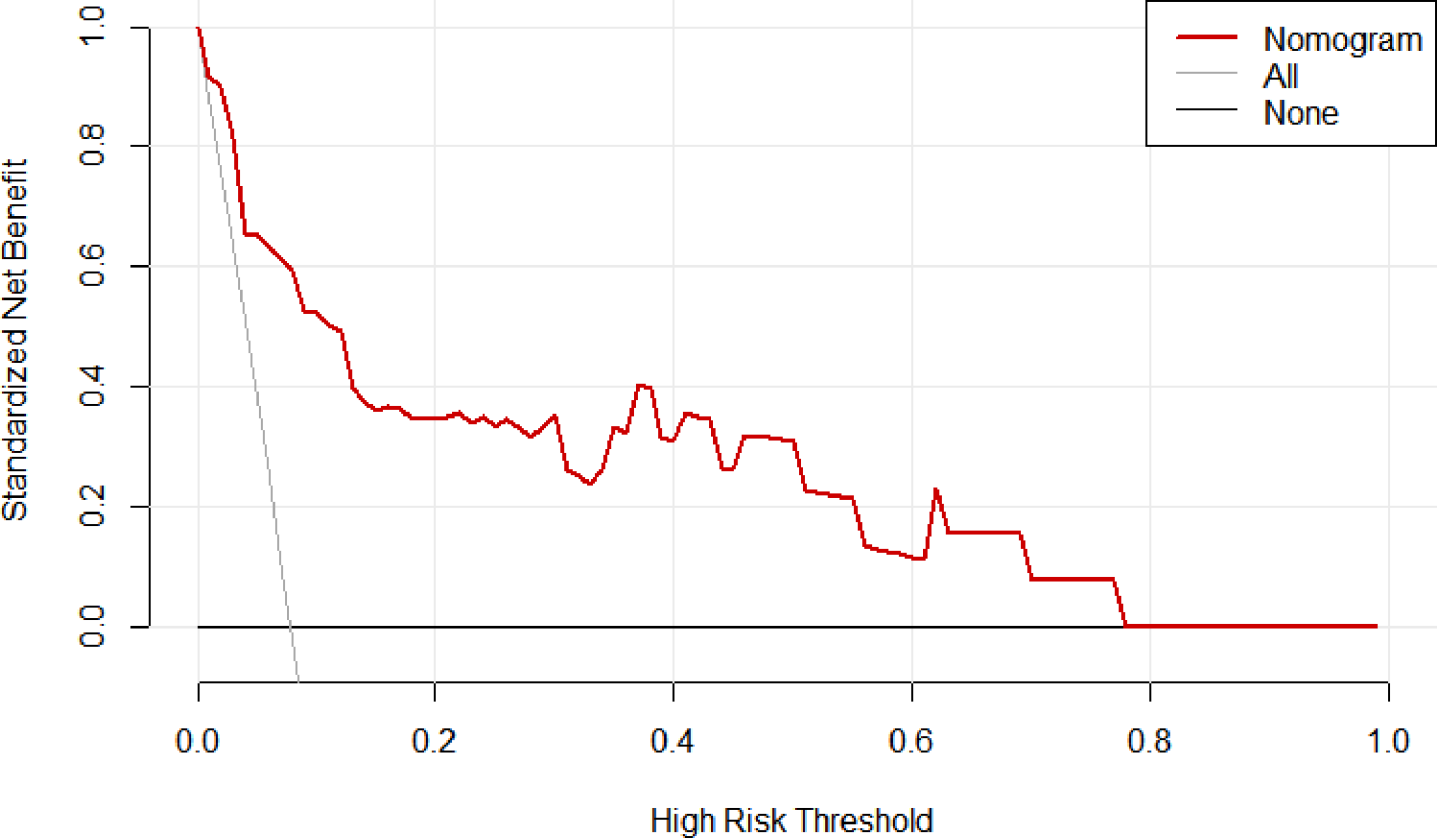
Decision curve analysis of the nomogram model.

**Fig 6:**
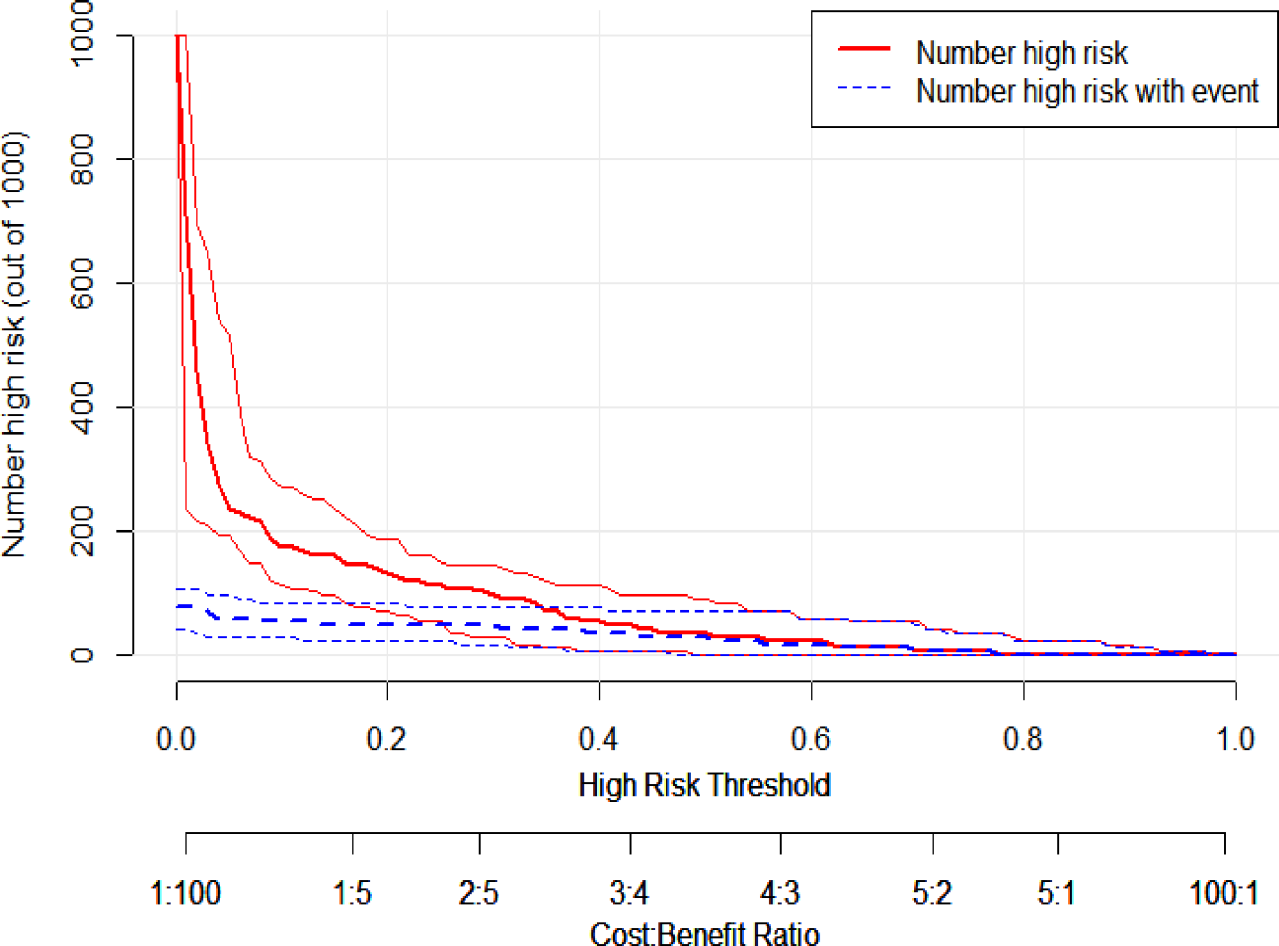
Clinical impact curve of the nomogram model.

The decision curve analysis in Fig. 5 shows that the nomogram provides significant net benefits for high-risk thresholds. The multivariate regression model is more advantageous in predicting CRKP infection in neuro-ICU patients, compared to targeting all patients or none. This is especially true for threshold probabilities ranging from 0 to 0.96, as revealed by the validation set. The clinical impact curve indicated a high degree of consistency between the predicted and actual distribution of the model (Fig. 6).

## DISCUSSION

This study has developed a nomogram model that accurately predicts the risk of CRKP infection in neurocritically ill patients. The model is based on easily obtainable indicators such as the number of antibiotics used, length of stay in neuro-ICU, and surgery. We validated the prediction model and found it to have good overall predictive value. Therefore, the established model could serve as an easy-to-use tool for predicting and preventing CRKP infection, which is a nosocomial infection with increasing incidence in neuro-ICU patients.

Our study found a prevalence of CRKP infection at 6.43%, which is consistent with the results of a previous study that reported a prevalence of 6.08% (29). However, recent studies have suggested that the prevalence of CRKP varies significantly across regions (15). For example, based on evidence from studies conducted in different regions of China, the prevalence of CRKP is less than 5% in Qinghai and Tibet, 10%–20% in Jiangsu and Zhejiang, and more than 20% in Beijing and Shanghai (30). In, addition, the prevalence of CRKP was high around the Yangtze River, spanning the East and Central regions of China (31). Previous studies have indicated that there is a greater prevalence of CRKP in regions with higher population densities and more advanced economies (32). This phenomenon can be attributed to the greater population density and lower air quality resulting from urbanization, which can make people more susceptible to bacterial infections (33). The recent study conducted in Beijing found multiple carbapenem-resistant genes in smog metagenomes (34). Also, with the continuous improvement of living standards, many patients prefer to go to tertiary hospitals for treatment, even for simple care needs (35). This results in a higher hospital bed density and, resultantly, a higher prevalence of CRKP (36). Therefore, medical institutions should attach great importance to regions where the prevalence of CRKP is increasing and take effective measures to control nosocomial infections caused by this superbug. It should be mentioned that most of the current research is limited to the eastern economic zone, the most economically developed region of China. The current status of CRKP infection in the central and western economic zones may be underestimated because of the lack of diagnostic tools and interventions.

In this study, neurocritically ill patients who used ≥2 antibiotics were found to be more likely to have CRKP infection. This finding supports the results of previous studies that have shown a significant correlation between the utilization of multiple antibiotics and the development of CRKP infection (37 – 39). The use of multiple antibiotics can lead to an increase in the risk of infection due to selective pressure on resistant microorganisms (40, 41). In clinical practice, the initiation of antimicrobial treatment for critically ill patients is mostly empirical (42). Considering the severity of critical illness, clinicians aim to achieve rapid clinical improvement and to avoid delay in treatment while waiting for etiological results (43, 44). However, previous studies have shown that the accuracy of empirical antibiotic use is still only 25.0%–49.5% (45). The most common conditions in which antibiotics are inappropriately used include prolonged treatment, treatment of non-infectious or non-bacterial syndromes, and the excessive use of broad-spectrum antibiotics (46). Research shows that structured workflow within the hospital affects the manner in which antibiotics are prescribed (47). Clinicians believe that the use of multiple and broad-spectrum antimicrobials reduces the risk of nosocomial infections and improves hospital performance measures (47, 48). Therefore, the epidemiological characteristics of infectious pathogens in local hospitals and wards should be fully considered in the early empiric administration to improve the accuracy of empiric administration. In addition, by improving the accuracy of pathogenic detection (e.g., alveolar lavage fluid samples) and timeliness (e.g., next-generation sequencing), pathogenic bacteria can be identified early and targeted antibiotic treatment given.

In this study, a long stay in the neuro-ICU was identified as an independent risk factor for CRKP infection. The transmission of resistant bacteria through air and direct contact in the neuro-ICU environment may contribute to the occurrence of nosocomial infections (29). With an increase in the length of hospital stay, neurocritically ill patients are exposed to an environment with more pathogenic bacteria for a long time, which further increases the risk of CRKP infection (49). Therefore, controlling the length of stay in neuro-ICU is a potential strategy for improving prognosis and reducing the risk of CRKP infection. Medical staff should implement early rehabilitation after assessing the condition of patients. After the condition of patients is relatively stable, they should be transferred to the general ward as soon as possible to reduce the length of stay in the neuro-ICU. In addition, a multidisciplinary diagnosis and treatment model should be established for neurocritically ill patients with multiple diseases (e.g., patients undergoing dialysis). Early treatment can facilitate recovery and shorten the length of neuro-ICU stay. Medical staff should isolate and treat patients who are at a high risk of developing CRKP infection as soon as possible. If necessary, these patients should be transferred to a single room to minimize the further spread of infection.

Surgery was identified as another independent risk factor for CRKP infection. Earlier studies have shown that surgery is a risk factor for CRKP infection (2, 50). Patients who have undergone surgical treatment in the neuro-ICU remain in a state of high stress and metabolism after surgery, accompanied by decreased immune function, which increases the risk of bacterial infections (51). Moreover, patients who have undergone surgery may require longer neuro-ICU stays and more invasive procedures, which in turn increases the risk of CRKP infection. Therefore, patients in the neuro-ICU who have undergone surgery should be closely monitored for nosocomial infections, especially CRKP, so as to prevent them as early as possible.

Based on the nomogram model developed in this study, we comprehensively interviewed the medical staff (*n* = 10, via convenient sampling) in the neuro-ICU to assess their opinions and expectations when using the model in clinical practice. Clinicians generally expressed a strong interest in the nomogram model and agreed with its importance in identifying patients at high risk for CRKP infection in routine clinical care. However, nurses expressed concerns about the complexity of the prediction model. Although similar barriers were identified in previous research (52, 53), studies to investigate the barriers in implementing the prediction models in actual clinical contexts are rare. Therefore, further research should also consider the way of integrating the prediction model into traditional clinical workflows. For example, this could involve forming an interdisciplinary team of medical staff and data scientists to create a detailed implementation plan for the model. Another overarching issue is that a minority of participants felt that the length of stay in the neuro-ICU in the model was not conducive to the early prediction of CRKP infection on hospital admission. The length of stay in neuro-ICU was not easily obtainable after admission, which may have hindered the model’s ability to assess quickly. A recent study suggested that a simple model with easily obtained variables would be more suitable and applicable in clinical practice (54). Therefore, in selecting future prediction model factors, it may be necessary to consider the time limit for obtaining predictors. Moreover, the development of an online web application, such as a risk calculator, may offer greater convenience for the application of prediction models in clinical settings. It may allow clinicians to identify high-risk patients and initiate prompt treatment to reduce the risk of CRKP infection.

### Strengths and limitations

This study is a significant contribution to the field, as it establishes the first nomogram model for predicting the risk of CRKP infection in patients admitted to the neuro-ICU. The nomogram has the advantage of using variables that can be obtained through routine examinations and has shown good discrimination and calibration in both the training and validation sets. However, this study has several limitations. First, our study was retrospective, which may introduce selection bias during data collection. Second, this study was conducted at a single hospital in one city with a limited sample size; therefore, the generalizability of the findings may be limited. In particular, the prevalence of CRKP can vary based on geographical, seasonal, and climatic factors, which may impact the applicability of our results to other regions. Therefore, our study could serve as a pilot study for larger, multiple center, and prospective studies, and future studies with multiple centers and larger populations from diverse areas should be carried out to confirm and expand upon our findings. Nevertheless, our findings are valuable and worth reporting as they focus specifically on neuro-ICU patients, a subset that has not been not widely investigated, and the evidence is rather needed for clinical practice.

### Conclusion

In this study, we developed a simple-to-use nomogram to predict the risk of CRKP infection in patients admitted to the neuro-ICU. The nomogram was constructed by integrating easily obtained variables, including the number of antibiotics used, surgery, and length of stay in neuro-ICU. This nomogram may serve as a useful tool for clinicians to design more effective treatment strategies for neurocritically ill patients with CRKP infection and to prevent other nosocomial infections. Although the medical staff showed a positive attitude toward the nomogram model, certain concerns remain to be addressed to facilitate the application of the model in clinical practice.

## Data Availability

The data sets used and/or analyzed during the current study are available from the corresponding author on reasonable request.

## SUPPLEMENTAL MATERIAL

Table S1 spectrum.03096-23-s0001.docx Definitions of the variables used in this study.

ASM does not own the copyrights to Supplemental Material that may be linked to, or accessed through, an article. The authors have granted ASM a non-exclusive, world-wide license to publish the Supplemental Material files. Please contact the corresponding author directly for reuse.
