# Supplementary material for: Development and application of a nomogram model for the prediction of carbapenem-resistant Klebsiella pneumoniae infection in neuro-ICU patients
Source: Microbiol Spectr. 2023 Dec 7;12(1):e03096-23. doi: 10.1128/spectrum.03096-23 (PMC10782973; doi:10.1128/spectrum.03096-23)
Supplement: Table S1 — Definitions of the variables used in this study. [file spectrum.03096-23-s0001.docx]

**Development and application of a nomogram model for the prediction of carbapenem-resistant *Klebsiella pneumoniae* infection in neuro-ICU patients**

**Supplementary material**

**Table S1.** The definition of the variables used in this study

| **Variables** | **Variable definition** | **The assignment** |
| --- | --- | --- |
| Age (years) | Age of the patient | Continuous variable |
| Male sex | Patient sex | Female = 0; Male = 1 |
| History of anticoagulants | Whether the patient had received any anticoagulants | No = 0; Yes = 1 |
| Length of stay in neuro-ICU (days) | Duration from neuro-ICU admission to discharge | Continuous variable |
| Diabetes | History of diabetes | No = 0; Yes = 1 |
| Hypertension | History of hypertension | No = 0; Yes = 1 |
| Heart disease | History of heart disease | No = 0; Yes = 1 |
| Cerebral infarction | History of cerebral infarction | No = 0; Yes = 1 |
| Chronic renal insufficiency | History of chronic renal insufficiency | No = 0; Yes = 1 |
| Admission season | Spring (March 1st to May 31st);  Summer (June 1st to August 31st);  Autumn (September 1st to November 30th);  Winter (December 1st to February 28th) | Spring = 1; Summer = 2; Autumn = 3; Winter = 4 |
| Admission diagnosis | Patient's diagnosis was made on admission | Cerebral haemorrhage = 0;  Brain injury = 1;  Aneurysm = 2;  Epilepsy = 3;  Brain tumour = 4 |
| INR | INR within 48 hours of admission | Continuous variable |
| BMI, (kg/m^2^) | BMI within 48 hours of admission | Continuous variable |
| Albumin, (g/L) | Albumin within 48 hours of admission | Continuous variable |
| Total protein, (g/L) | Total protein within 48 hours of admission | Continuous variable |
| RBC, (10^12/L) | RBC within 48 hours of admission | Continuous variable |
| WBC, (10^9/L) | WBC within 48 hours of admission | Continuous variable |
| Platelets, (10^9/L) | Platelets within 48 hours of admission | Continuous variable |
| Hemoglobin, (g/L) | Hemoglobin within 48 hours of admission | Continuous variable |
| Serum creatinin, (umol/L) | Serum creatinin within 48 hours of admission | Continuous variable |
| Procalcitonin, (ng/ml) | Procalcitonin within 48 hours of admission | Continuous variable |
| ALT, (U/L) | ALT within 48 hours of admission | Continuous variable |
| AST, (U/L) | AST within 48 hours of admission | Continuous variable |
| Uric Acid, (umol/L) | Uric Acid within 48 hours of admission | Continuous variable |
| Urea, (mmol/L) | Urea within 48 hours of admission | Continuous variable |
| LAC, (mmol/L) | LAC within 48 hours of admission | Continuous variable |
| Glucose, (mmol/L) | Glucose within 48 hours of admission | Continuous variable |
| Systolic pressure, (mmHg) | Systolic pressure within 48 hours of admission | Continuous variable |
| Surgery | Whether patients had surgery before CRKP infection occurred | No = 0; Yes = 1 |
| Number of antibiotics* | Number of antibiotic used by patients prior to the onset of CRKP infection | ≥2 = 1; <2 = 0 |

Note: INR, International Normalized Ratio; BMI: Body mass index; RBC, red blood cell; WBC, white blood cell; ALT, Alanine transaminase; AST, Aspartate transaminase; LAC, Lactic acid. *:According to the Guiding Principles for the Clinical Application of Antibiotics, infections that can be effectively treated by a single drug do not require combined use of drugs (1). Severe infections that cannot be controlled by a single antibacterial drug and infections caused by multi-drug-resistant bacteria or pan-drug-resistant bacteria usually use a combination of two drugs (1). Moreover, we also realized previous studies have concluded that patients with multidrug-resistant infections with more than two risk factors were recommended to receive two or more antibiotic treatment regimens (2,3).

References

1. The clinical application of antibacterial drugs guiding principles revised working group. Clinical application of antibacterial drugs guiding principles: 2015 edition. 6. Vol. 8. People’s Health Publishing House; 2015.

2. Cillóniz C, Torres A, Niederman MS. 2021. Management of pneumonia in critically ill patients. Bmj 375:e065871.

3. Maruyama T, Fujisawa T, Ishida T, Ito A, Oyamada Y, Fujimoto K, Yoshida M, Maeda H, Miyashita N, Nagai H, Imamura Y, Shime N, Suzuki S, Amishima M, Higa F, Kobayashi H, Suga S, Tsutsui K, Kohno S, Brito V, Niederman MS. 2019. A Therapeutic Strategy for All Pneumonia Patients: A 3-Year Prospective Multicenter Cohort Study Using Risk Factors for Multidrug-resistant Pathogens to Select Initial Empiric Therapy. Clin Infect Dis 68:1080-1088.
